# Health capital: toward a conceptual framework for understanding the construction of individual health

**DOI:** 10.1057/s41285-020-00145-x

**Published:** 2020-06-23

**Authors:** Anna Schneider-Kamp

**Affiliations:** Department of Marketing & Management, University of Southern Denmark, Campusvej 55, 5230 Odense M, Denmark

**Keywords:** Health capital, Bourdieu’s forms of capital, Individualization of healthcare, Human capital theory

## Abstract

Emerging perspectives of health as individualized and privatized capital seem promising to shed light on the construction of individual health in the face of the growing individualization of healthcare. This article reviews extant perspectives of health as capital, reflecting upon how a conceptualization of *health capital* might be conceived by two of the main contrasting traditions: human capital theory affiliated with the Chicago School of Economics and Bourdieusian concepts of social field and capital. Arguing that a Bourdieusian perspective is potentially more fruitful to capture the importance of social and cultural dimensions in the construction of individual health, this article arrives at a conceptualization of ***health capital*** as the *aggregate of the actual or potential resources possessed by a given agent that have the capacity to affect the position of agents in the social field of health.* Drawing on Bourdieu’s conceptualization of forms of capital, this article discusses the efficacy, the legitimation, and the positioning of health capital, uncovering its potential for understanding contemporary trends in health practices and health discourse.

## Introduction

The Renaissance saw a rediscovery of the ideals and medical practices of ancient Greece (Kleisiaris et al. 2014), guiding the medical profession toward a focus on scientific evidence and university-based training of physicians. The medical profession’s responsibilities once more extended into societal matters of public health, personal hygiene, and social inequality as well as individual matters of healthy lifestyles (Cipolla 1976). The accelerating growth of medical evidence and abilities in the nineteenth and twentieth century and the ensuing further expansion of the medical profession’s responsibility (Illich 1976) have become objects of “medical social science research on topics as patient autonomy, social equality, and power relations in specific contexts of care or in entire medical systems” (Kaufman 1988, p. 338). Medical sociologists have voiced concerns regarding the medicalization of everyday life (Zola 1972), where non-medical conditions become subjects of diagnosis, treatment, and prevention and, consequently, the objects of social control (Conrad 1992).

While historically, medical professionals were predominantly carrying the burden of responsibility for health, toward the twenty-first century, responsibility for health is also increasingly becoming a matter of individual concern on a mass scale. Health policies have begun to assign some degree of responsibility for health to patients in attempts to empower them through self-care and shared decision-making (Fumagalli et al. 2015). In addition, Rose (1999, p. 88) observes a fusion of “responsible citizenship” with “individuals’ projects for themselves,” transforming medical responsibility from “a social norm” into “a personal desire” and health from a matter of course into a status symbol. Crawford (1977) argued that responsibility for one’s health is driven by an ideology of victim blaming and has given birth to a mass culture of “healthism,” which is preoccupied with “health as a primary – often the primary – focus for the definition and the achievement of well-being” (Crawford 1980, p. 368), is pervading society.

These developments are profoundly affecting the construction of individual health. Shim (2010, p. 96) describes how the “cultural resources that patients bring to the health care encounter” are empowering them to participate to a higher degree than previously in the construction of their health. Contemporary research on online health communities (Kingod et al. 2017) suggests that such illness networks provide social support extending beyond professional medical care. Schneider-Kamp and Askegaard (2019) suggest a patient-centered perspective on empowerment as “emerging from a bricolage of tactical interactions with social environments” and highlight how the transformation of society into an information and consumer society is contributing to empowering some of the more resourceful individuals to even entirely take over the construction of their health.

The construction of health, thus, seems to increasingly depend on the availability and types of resources at the disposal of the individual, encouraging a view of health as individualized and privatized “capital.” This article aims to develop the notion of health capital to aid in the understanding of the construction of individual health. First, it reviews existing perspectives of health as capital as well as the theories of human, social, and cultural capital underlying these perspectives. Then, it reflects upon how a conceptualization of health capital could be conceived by two contrasting traditions: human capital theory and Bourdieu’s forms of capital. Finally, it discusses the efficacy, the legitimation, and the positioning of health capital from a Bourdieusian perspective, uncovering its potential for understanding contemporary trends in health practices and health discourse.

## Health as capital

The idea of relating the health of an individual to capital goes back to debates in political economy starting with Mushkin’s (1962) view of health as an investment and Becker’s (1964) view of health as a part of human capital. Grossman (1972) builds on these perspectives and introduces the term “health capital” as part of a model of the demand for the commodity “good health.” This model views health as a “durable capital stock that produces an output of healthy time” (Grossman 1972, p. 246) and depreciates with age, but that can be invested in through medical treatments.

Grossman (1972) argues for health capital being different from other forms of human capital: instead of focusing on increasing worker productivity, health capital focuses on life-long earnings by viewing health as both a “consumption commodity” directly entering consumers’ “preference function” and an “investment commodity” resulting in a larger amount of time available for “producing money earnings and commodities” (Grossman 1972, p. 225). Sick days incur an “opportunity cost of the time that must be withdrawn from competing.”

Grossman’s (1972) health capital and the associated model of the demand for health have spawned one of today’s main recognized paths of research into the economics of health. The field of health economics is predominantly focused on studying the functioning of the healthcare system and ways of improving health policies. Grossman’s model and its successors provide numerical tools for studying individual health from a statistical perspective, where the individual is a tiny mechanical part of the macro-level healthcare apparatus.

The human capital perspective on health as capital is more recently being supplemented by Turner’s (2003, p. 4) social capital perspective with its focus on social capital as the main determinant of health, arguing that “the quantity and quality of a person’s social relationships and social networks play an important part in the maintenance of their health, and at the same time provide resources for their recovery from illness.” Turner draws predominantly on Durkheim’s (1897) study of suicide as a sociological rather than an individual psychological phenomenon.

Turner reviews social capital theory as it regards health and illness, making a case for integrating Durkheim’s analysis of social reality into contemporary medical sociology. Following up on Parsons’ (1951) attempts to establish a sociology of sickness, Turner (2003, p. 17) argues that “social density” is the “underlying cause of health,” and its absence is linked to an increase of illness. Consequently, he views social capital theory as “the most promising sociological, as opposed to social psychological, anthropological or cultural, account of health and illness that we have” (Turner 2003, p. 4).

The human and social capital perspectives on health as capital are being further supplemented by a cultural perspective. Shim (2010) introduces the notion of “cultural health capital” as a theoretical framework for understanding the dynamics of the interaction of patients with health professionals in the context of the study of health inequality. Her concept draws upon Bourdieu’s (1986) notion of “cultural capital,” which he distinguishes from “economic” and “social” capital.

Shim aims to explain the role of cultural differences at the societal level in concrete encounters between patients and health professionals by conceptualizing “cultural health capital as a specialized form of cultural capital that can be leveraged in healthcare contexts to effectively engage with medical providers” (Shim 2010, p. 3). This gives rise to a definition of “cultural health capital” as “a specialized set of cultural skills, behaviors and interactional styles that are valued and leveraged as assets by both patients and providers in clinical encounters” (Dubbin et al. 2013, p. 114). This relational interpretation of Bourdieusian cultural capital is reminiscent of Prieur and Savage’s (2011) understanding of cultural capital, which they argue continues to be relevant to a dynamic society when viewed as a relative rather than an absolute concept.

These emerging views of health as capital have their roots in the ideas of the classical economists and sociologists of the eighteenth and nineteenth centuries. Grossman’s (1972) concept of health capital is built on top of Becker’s (1964) “human capital,” a concept that can ultimately be traced back to Smith’s (1776) ideas on “capital stocks.” Turner discusses his sociological perceptive on health as capital on the general backdrop of social capital theory as presented by Lin (2001) and in relation to Coleman’s (1988) notion of social capital, which, in turn, is also rooted in Becker’s (1964) human capital. Lin integrates political scientific concepts from Tocqueville (1831) to Putnam (1993) with medical sociological ones such as Parsons’ (1951) sick role.

Both Turner and Shim draw heavily upon Bourdieu’s (1986) distinction of forms of capital, in particular, “social capital,” which is based on relationships and affiliations, and “cultural capital,” which is based on cultural assets such as skills and knowledge. Bourdieu’s work on capital is, in turn, influenced by Marx’s (1847) preference for social relations over interactions, by Weber’s (1921) work on authority through domination and class analysis, and Durkheim’s (1897) ideas on the reproduction of social structures.

**Fig. 1:**
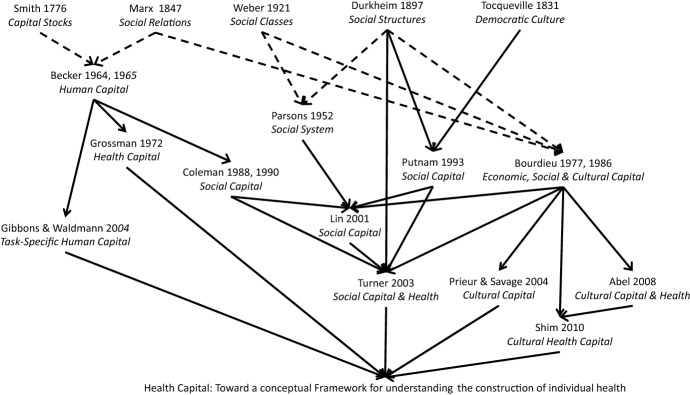
Mapping of research perspectives on health as capital underlying the conceptualization of health capital in this article

The map in Fig. 1 visualizes the historical paths toward viewing health as capital relevant to the theoretical considerations of this article. In this figure, regular arrows indicate explicit engagement while dashed arrows indicate influential academic traditions.

## Rethinking health capital

There are two contrasting theoretical traditions through which a notion of health capital for understanding the individual construction of health could be conceptualized: human capital theory in the tradition of the Chicago school of economics and Bourdieu’s work on the forms of capital.

### Human capital theoretic perspective

Through a human capital theoretic perspective, such a conceptualization of health capital could be viewed as a capital stock of dual nature, one that could be an input as well as an outcome of production processes. When health capital is viewed as an input, any investment of individuals into their health capital would be made with the goal of using it as an asset in obtaining material returns through the production of healthy time (Grossman 1972). Grossman would refer to health capital in this sense as an investment commodity. While health capital clearly could be an asset in the production of market goods and other commodities, the construction of individual health could also be viewed as an end in itself. In this sense, it could be viewed as the outcome of the production processes of the consumption commodity health capital driven by the “fundamental demand for ‘good health’” (Grossman 1972, p. 248). The duality of health capital would rest on the conversion of different forms of human and economic capital within standard market forces, i.e., through the exchange of money, market goods, and commodities.

Human capital theory also has a long tradition of considering specific forms of human capital: from general-purpose and industry-specific to firm-, occupation-, and task-specific human capital (Gibbons and Waldman 2004). Task-specific human capital takes up Smith’s (1776) view on task-specific specialization as a driver of productivity and is based on the idea that “much of the human capital accumulated on the job is due to task-specific learning by doing” (Gibbons and Waldman 2004, p. 203). Health capital as a conceptual framework for understanding the construction of individual health could be viewed as task-specific at different scales: from tasks such as the patient-specific day-to-day management of chronic illnesses like diabetes (Scambler et al. 2014) to more general tasks such as the prevention of lifestyle diseases through individual exercise and dieting advocated by health promotion campaigns (Ayo 2012).

Human capital theory often draws upon a homogeneity assumption as, e.g., formulated by Stigler and Becker (1977, p. 76): “rules and tastes are stable over time and similar among people.” Nevertheless, in regard to health, there is an acknowledgement that, on the input side, the effectivity of the process of producing good health depends on “certain environmental variables” such as “the level of education of the producer” (Grossman 1972, p. 225). Likewise, on the output side, the investment in health is associated with side effects, often referred to as positive externalities in the literature on human capital theory (Ciccone and Peri 2006). These environmental variables, positive externalities, and other external factors are inherently outside the scope of human capital theory (Tan 2014), a priori limiting the potential value of a conceptualization of health capital through human capital theory.

### Bourdieusian perspective

A conceptualization of health capital from a Bourdieusian perspective would have the potential to capture such factors through the concept of social fields, which embed the rules, the agents, and relations of a particular domain of activity (Bourdieu 1984). The agents strive for social distinction within the field, accumulating symbolic capital in the form of recognition and status. This symbolic capital complements the economic, social, and cultural capital that shapes their class belongingness. Class and status, in turn, structure the agents’ capacities and dispositions. Bourdieu (1986) views capital as accumulated labor that has the capacity to affect the social positions of agents in a field.

Bourdieu and Wacquant (1992) introduce the notion of “field-dependency” of capital to signify how capital determines the social positions of the agents within a given field depends on the specific rules and relations of that field. This could open up for understanding health capital as field-dependent capital, determining the social positions of the agents within the social field of health. Here, the social field of health includes not only institutionalized health encounters in the form of interactions between patients and health professionals but also individuals’ everyday health practices outside of institutionalized contexts. Health capital could then be viewed as a unique form of capital different from but drawing upon the synergy of economic, social, cultural, and symbolic capital contextualized to the social field of health.

Economic capital plays a significant role in constructing individual health and shaping everyday health practices. Besides the purchase of medical services or products when one faces a medical condition, economic resources affect whether and how one implements a healthy lifestyle, e.g., through the consumption of (super)foods perceived to offer health benefits (Kamiński et al. 2020) or through personal trainers and self-tracking (Pantzar and Ruckenstein 2015). Unsurprisingly, statistics indicate a significant positive correlation between economic capital and life expectancy (Chetty et al. 2016).

Social capital contributes likewise to the construction of individual health. Family members and close personal acquaintances have always played a significant role in everyday health practices. Individual health has been found to depend on both the health and the health attitudes of family members (Jacobson 2000). Social capital in the form of strong social relations is correlated with good health (Turner 2003), in general, and longer life expectancy (Kennelly et al. 2003), in particular. Another important source of health-related social capital is patient associations, where sufferers unite in order to support each other (Carlsson et al. 2006), and online health communities, which offer connectivity and social support independent of geographical and class belongingness (Kingod et al. 2017).

Cultural capital plays a significant role in understanding how culture shapes health inequality (Dubbin et al. 2013; Shim 2010) and health-related help-seeking practices (Doblytė 2019). Cultural embeddedness, whether innate or acquired through media exposure, may open up access to alternative medical services or products (Thompson and Troester 2002). Prieur and Savage (2011) argue that cultural capital is to some degree dependent on the cultural context, with a bias toward a global orientation. Schneider-Kamp and Askegaard (2019) show how more general and globalized competences and attitudes play at least as important a role in the construction of individual health as more locally contextualized cultural aspects.

A general attitude of self-efficacy (Bandura 1977) combined with health information-seeking behaviors (Weaver et al. 2010) is providing a natural ground for the proliferation of “health literacy” or “the degree to which individuals have the capacity to obtain, process, and understand basic health information and services needed to make appropriate health decisions” (Ratzan and Parker 2000, p. 147). This form of cultural capital becomes embodied over time and encourages an “ethic of self-conduct,” where individuals are viewed as “competent” if they are successfully “acquiring the skills and making the choices to actualize” themselves (Rose 1999). In the context of mental health services, initiatives built on the idea of expertise by experience actively encourage the accumulation of such cultural capital (Toikko 2016).

While Shim’s (2010) cultural health capital focuses on the role of culture in health encounters, Doblytė (2019, p. 287) argues that both cultural health capital and social capital that “can be converted into cultural health capital” are crucial in the study of mental health services. A conceptualization of health capital that integrates forms of capital other than cultural capital would allow for reflecting upon how, for example, social capital in the form of social embeddedness influences the construction of individual health without having to consider its conversion into cultural capital.

The relevance of taking multiple forms of capital into account when studying health is already described by Abel (2008, p. 1), who finds that “class related cultural resources interact with economic and social capital in the social structuring of people's health chances and choices.” A Bourdieusian perspective, thus, could offer a holistic view on the synergy of the forms of capital and allow for capturing the social and cultural embeddedness of contemporary health practices and health discourse. It could provide not only an opportunity for a deep investigation of the *how* but also the potential to explain the *why* in the construction of individual health by considering the symbolic aspects of capitals.

Bourdieu (1987, p. 4) defines symbolic capital as “the form that the various species of capital assume when they are perceived and recognized as legitimate.” While social and symbolic capital are inherently “very strongly correlated” (Bourdieu 1987, p. 4), Bourdieu (1986, p. 49) also postulates that the “transmission and acquisition” of cultural capital “are more disguised than those of economic capital” and that “it is predisposed to function as symbolic capital.” By this, he refers to cultural capital often not being recognized as capital, but as “legitimate competence, as authority exerting an effect of (mis)recognition.”

Integrating symbolic capital into a conceptualization of health capital could allow for a more nuanced understanding of the rationale behind individual practices of healthcare. Participation in an online health community could not only be viewed as a matter of obtaining social support but also as a quest for being recognized as legitimate sufferers. Expertise by experience could be understood not only in terms of accumulation of relevant health knowledge but also as a means of social distinction within the relevant social structures. Consuming superfoods, expensive training shoes, carbon fiber bicycles, luxury wearables for self-tracking, and the services of personal trainers could be seen as driven by a desire for social distinction and, ultimately, status rather than as an investment in physical well-being. In this spirit, healthism as a neoliberal project and a state ideology on the societal level could be nuanced by an understanding of the agents’ quest for social distinction not only the social field of health but also in other fields.

Symbolic capital could also help to understand seemingly contradictive and even counterproductive practices. For some young men, status based on perceptions of masculinity outcompetes concerns for health, reducing their interactions with health services (Gough 2006). For similar reasons, middle-aged men routinely and purposively damage their joints and actively increase their risk of cardiovascular diseases by training for and competing in marathons (Schwartz et al. 2014). Likewise, women exhibit a fair share of self-destructive behaviors such as eating disorders and exercise addiction (Lichtenstein et al. 2017) in their quest for the “perfect body” and the status that this ideal conveys.

Embracing a Bourdieusian perspective, this article proposes to conceptualize ***health capital*** as the *aggregate of the actual or potential resources possessed by a given agent that have the capacity to affect the position of agents in the social field of health.* Health capital encompasses the field-dependent skills, competencies, social relationships, financial means, and status that can, immediately or mediated through conversion from other forms of capital, be employed toward the preservation of good health and the management of illness. It, thus, draws upon the synergy of and complements economic, social, cultural, and symbolic forms of capital. In the remainder of this article, unless explicitly otherwise noted, the term “health capital” will refer to this conceptualization.

Such a Bourdieusian conceptualization of health capital appears to be potentially most fruitful in understanding the construction of individual health as it allows transcending the input–output-oriented production focus of human capital theory by emphasizing the causal and human relations underlying individual healthcare practices. The following sections will use this perspective to explore and discuss the efficacy, the legitimation, and the proper place for health capital through reflection on socially and culturally embedded mass-cultural trends in the increasingly complex and individualized social field of health.

## The efficacy of health capital

Bourdieu (1986, pp. 53–54) discusses the efficacy of different forms of capital, postulating that social and cultural capital “can be derived from economic capital, but only at the cost of a more or less great effort of transformation, which is needed to produce the type of power effective in the field in question.” The effort required to leverage economic capital as health capital and, thereby, the efficacy of health capital is likewise field-dependent, as the rules of the field and the dispositions of the agents differ significantly from one context to another. One would, for example, expect a higher degree of convertibility in predominantly privately organized healthcare systems such as the one of the United States compared to tax-financed egalitarian systems such as the Danish one, where healthcare is virtually free and the market for privately paid medical services only fills a small niche (Saunders 1995).

Efficacy as the ability to produce desired results is inherently dependent on the nature of this result. While no amount of health capital (currently) can produce the result of curing chronic conditions such as Parkinson’s disease, Duncan and Earhart (2011) describe how social capital in the form of participation helps patients suffering from Parkinson’s disease in the management of their condition, with the result being an improvement in their quality of life. The Paralympics do not only motivate individuals with disabilities to accumulate health capital but also allow them to achieve social distinction as a secondary result by gaining considerable prestige (Gold and Gold 2007). While the conceptualization of health capital demarcates resources according to their capacity to preserve good health and manage illness, agents employ these same resources to produce a wider range of desired results.

The efficacy of health capital further depends on individuals’ abilities to leverage their health capital. Health-related knowledge, competences, and skills are embodied over time and contribute to an “individual heritage of dispositions” (Lahire 2003), one that is slowly but constantly changing as the agents continue to accumulate and embody further health capital as well as dispositions regarding the use of information and communication technologies (Jacobs et al. 2017). The entailing increase in self-efficacy and empowerment is at odds with the dominating force relationships in the healthcare field, which are built on institutionalized professional care and the expert status of health professionals. Bourdieu (1986) uses the term “hysteresis" for such a situation where dispositions and field are out of sync. As a result of hysteresis, he postulates a struggle for domination, which is challenging and ultimately reshaping the field, eventually alleviating the hysteresis.

An example of such a struggle for domination and the consequent challenging of the field has been observed in the study of everyday health practices, where patients do not have the power to directly change the rules of the healthcare field but use a number of tactics to circumvent them (Schneider-Kamp and Askegaard 2019). As these tactics become embodied, these new dispositions change the demands placed on health providers by both patients and policymakers, slowly changing the rules of the field and alleviating the hysteresis caused by the changing dispositions.

The COVID-19 crisis of 2019/20 provides another, more extreme example of hysteresis, where the attempts to contain the pandemic have rapidly changed all the rules of the field within a matter of weeks. Much of embodied cultural capital in the form of long-lasting dispositions has suddenly become irrelevant—with the possible exception of proper hand washing routines. The efficacy of objectified cultural capital in the form of documented experience and medical knowledge has likewise become questionable in the light of the lack of knowledge and the uncertainty pervading the unfolding of the pandemic. Social distancing is severely affecting the appropriation and, thereby, the efficacy of social capital toward the preservation of well-being and, ultimately, mental health (Zhang et al. 2020). The impending overload of the healthcare system on a global scale is even affecting the efficacy of economic capital as numerous countries have chosen to unconditionally postpone non-essential treatments for the duration of the pandemic.

## The legitimation of health capital

One way for individuals to legitimate their health capital is by verifying it through the medical authority of trained health professionals with their knowledge, in turn, legitimated by both their educational degrees and the positions they occupy in institutionalized healthcare (Abel 2008). Here, the power relations are to a large degree based on symbolic capital, i.e., the capital that is based on recognition and legitimates agents in the field of institutionalized healthcare.

Furthermore, while this field is a large and important sub-field of the social field of health, ultimately, it is only one of many. Bourdieu and Wacquant (1992) consider fields that are constituted of “sub-fields” with competing and mutually incompatible inherent power relations. They argue that the legitimacy of capital in such fields can depend on the sub-field under consideration, as each of them has the potential to offer different ways of legitimating health capital as discussed below for two prominent examples of sub-fields.

First, consider the sub-field of the commoditized health market (Henderson and Petersen 2002) with the individual in the role of the demanding consumer and health professionals as well as pharmaceutical and medical device companies in the roles of providers of medical services and products (Lee 2015). Here, the mere availability of products and services already contributes to their perception as being legitimate. The availability of alternative medical products (Thompson and Troester 2002) through online retailers contributes to the legitimation of the application of alternative medicine to preserve health or recover from illness.

Second, consider the sub-field of internet health, where the broad availability of online medical information (Jacobs et al. 2017) and the existence of online health communities (Kingod et al. 2017) legitimate individuals’ health capital. The public availability of online manuals for medical doctors can be seen as legitimating their use for self-diagnosis and self-treatment while online health communities legitimate health capital by offering individuals to be recognized as legitimate sufferers, even in situations where health professionals cannot or do not want to do so (Schneider-Kamp and Kristensen 2019).

In general, we see a diversification of the structures that legitimate health capital. Legitimation by the medical authority of trained health professionals is supplemented by, e.g., legitimation through market mechanisms and internet health. This diversification of structures of legitimation leads to a diversification of norms. While the norms in the social field of health traditionally have been set by the health profession based on “expert knowledges” Lupton (2013), alternative norms can also be set by, e.g., online health communities based on the lay knowledges of their members. These alternative norms are legitimated by the “counter-expertise” of “proto-professionals,” i.e., of “lay persons” who “have become experts in redefining their every troubles as ‘problems amenable to treatment by this or that profession’” (Rose 1999, p. 87).

The legitimation by proto-professional peers in online health communities has shown potential to increase the health capital of its members through “social support and connectivity” and “experiential knowledge sharing” (Kingod et al. 2017, p. 89). There are grounds for concern, though, when individuals are only exposed to information from like-minded peers and simultaneously shielded from more mainstream information sources. The legitimation of health capital by proto-professional peers within such a “filter bubble” (Pariser 2012) has, for example, given rise to the phenomenon of anti-vaccination movements (Goldenberg 2019), which is challenging public health on a global scale.

## A proper place for health capital

The conceptualization of health capital proposed in this article extends Shim’s (2010) cultural health capital, which aims to explain inequalities in institutionalized healthcare with a focus on how cultural capital provides advantages to patients in encounters with health professionals. First, health capital does not presuppose an institutionalized context such as health encounters but also provides a means for understanding everyday health practices that do not involve health professionals in prominent roles or at all. Second, by integrating social and economic resources and symbolic aspects on an equal footing with cultural ones, health capital allows exploring their effect without having to consider them mediated through transformation to cultural capital. While cultural capital in the form of medical knowledge and communication skills are naturally dominant in health encounters, other forms of capital such as social capital in the form of social density (Turner 2003) play a significant role in a broad spectrum of everyday health practices.

The proposed conceptualization of health capital encourages policymakers to be aware of the complexity of multiple forms of health-related resources and assets possessed and employed by individuals in the construction of individual health. Health policies can then focus on improving the alignment between the rules of the healthcare field and individuals’ dispositions, strengthening the link between “public objectives for the good health and good order of the social body with the desire of individuals for personal health and well-being” (Rose 1999, p. 74). Health capital has the potential to explain how “macro-level phenomena” are “manifested and actualized in lived experience and the day-to-day unfolding of social life” as well as how “micro-level interactions accrete and constitute larger-scale social processes and structures” (Shim 2010, p. 1).

An example of how macro-level phenomena are actualized in lived experience can be found in the debate on the mixed success of patient empowerment initiatives. Greener (2008), as well as Winthereik and Langstrup (2010), identify a lack of resources as a factor inhibiting the success of state-initiated patient empowerment initiatives in the United Kingdom and Denmark, respectively. Health capital allows understanding the observed heterogeneity by considering that individuals differ regarding their health capital and, consequently, perform widely varied everyday health practices and react differently on efforts to strengthen their health literacy and self-efficacy. Instead of targeting patients exclusively by shared medical conditions—as customary in empowerment initiatives—taking individuals’ social, cultural, and economic background into account has the potential to improve the outcome of such initiatives.

Ultimately, the practices of individuals are always aligned with their self-interests through an “economy of the proper place” as summarized by de Certeau’s (1984, p. 55):

In sum, these practices are all dominated by what I shall call an economy of the proper place (une économie du lieu propre). In Bourdieu’s analysis, this economy takes two forms, equally fundamental but unarticulated; on the one hand, the maximisation of capital (material and symbolic wealth) that constitutes the essence of patrimony; on the other, the development of the body, both individual and collective, that generates duration (through its fertility) and space (through its movements).

Health capital is subjected to both forms of this economy: health capital is inherently linked to the development of the body and the maximization of health capital contributes to social recognition and status in a variety of fields. By its ability to capture this duality, health capital as discussed in this article provides a conceptual framework for the construction of individual health that is broadly applicable to the analysis of contemporary trends in health practices and health discourse.
